# Know Where You Stand: Affective Effects of Becoming Aware of a Place's National Socialist History

**DOI:** 10.3389/fpsyg.2022.936621

**Published:** 2022-09-14

**Authors:** Melissa Ries, Stephan Schwan

**Affiliations:** Realistic Depictions Lab, Leibniz-Institut für Wissensmedien (IWM), Tübingen, Germany

**Keywords:** historic place, historic site, history awareness, National Socialist, affective judgment, mood

## Abstract

Visiting historical places can give important impulses regarding education of history, society, and politics. While there does exist extensive research on visitors' experiences at memorial sites, little is known about the impact of everyday places holding dark history. Two experimental studies took place in a research institute, a former women's clinic, where in the time of National Socialist (NS) dictatorship in Germany hundreds of forced sterilizations took place. Historical awareness was manipulated *via* systematic variation of prior information. We found partial evidence that historical awareness had a negative effect on personal mood. Awareness of the site's NS history had a negative effect on the perceived valence of related photos but did not influence their qualitative description. Also, there was partial evidence that the site itself was perceived less positively and evoked more arousal when participants were aware of its NS history. Possible reasons are discussed.

## Introduction

In *terms of lieux de memoire*, Nora (1989) stated that a variety of things can function as a center point for a collective, identity-forming memory, not only for certain persons or events in time but also for historic places. Accordingly, Manzo (2003) noted that “the dominant political culture influences the appearance, meanings and uses of space” (p. 55), and we might add that this also holds for what is perceived and remembered by visitors. Therefore, while historic places often hold references to different events in time, it depends on the dominant political culture (and the historical expertise of the visitor) whether anything is actually going to be remembered, and if so, what. *Deutsche Erinnerungsorte* (Francois and Schulze, 2001) may be seen as a German counterpart to Nora's work. However, while there are chapters about Auschwitz and memorials, the work lacks references to everyday places. Therefore, history in the immediate vicinity and subtle traces of this history remain largely hidden. In contrast, the *dig where you stand* movement (Lindqvist, 1978) exemplifies an approach that focuses on places of memory in everyday life rather than on popular heritage sites. It can be described as a network consisting of several autonomous and diverse groups attempting to transform the prevailing memory politics by persistently achieving many small changes, such as changing street names referring to the Nazi period (Wüstenberg, 2017).

This twofold aspect of collective memory, both at “Erinnerungsorte” in Nora's sense and at everyday places, plays a major role for Germany's dealing with the crimes of the National Socialist (NS) regime, taking much effort to make people aware of the historical dimension of NS related places. Within the NS period (1933–1945), a countless number of War Crimes took place. These crimes include, among others, the *Holocaust*, that is, the mass murder of about six million European Jews, the persecution and murder of political dissidents, the so-called *Aktion T4*, namely, the systematic murder of people with mental and physical disabilities, as well as the forced sterilization of so-called “racially inferior” people. The origins of German eugenics can be traced back to the 1890s (Weiss, 1987). However, the racial ideology found its peak in Germany during the NS period when Hitler's regime passed the *Law for the Prevention of Offspring with Hereditary Diseases*. The idea behind the “German eugenics” and the so-called “race hygiene” was based on the assumption that mankind could be divided into inferior and superior races, whereas persons considered to be “superior” (persons having “Nordic” or “Aryan” traits) were encouraged to produce a large number of offspring, while on the other hand persons considered to be “inferior” (for example, persons having mental or physical disabilities, having homosexual, intersexual or transgender tendencies, being classified as so-called “racial inferior,” which meant featuring any other than the “Aryan” trait) were requested to be sterilized voluntarily, but often these procedures were performed against the concerned person's will (for comprehensive information on this topic see Burleigh, 1994; Bayer, 2008).

Providing memorials, museums and documentation centers at places which are related to the NS history is based on the assumption that at such sites informing and educating about historical events is particularly effective. Accordingly, memorials and documentation centers are frequently visited during school field trips and report large annual numbers of visitors, for example, 900.000 visitors in 2018 at the Concentration Camp (Konzentrationslager; KZ) memorial Dachau, 700.000 at the KZ memorial Sachsenhausen, and 500.000 at the KZ memorial Buchenwald (Das Gupta and Sandkuhl, 2019).

While the historical dimension is evident at sites that host KZ memorials or documentation centers, there are also sites that are not utilized in such ways and therefore often not identifiable as historical sites as such. Among others, this may be either because there is hardly any substance or structure of the former building left or because the building has been renovated and modernized over the years and now holds a completely different function. Methods to raise the awareness of the historical dimension of such everyday places in Germany range from so-called *Stolpersteine*^1^ (so-called ‘stumbling blocks' along the sidewalks, having a brass plate engraved with the names and personal life data of persons who used to live there before they were deported and often killed by the NS regime.) to interpretation panels which usually combine text explanations with historical photos of the site.

Visitors of KZ memorials or NS related documentation centers have usually deliberately chosen to visit these sites, are aware of the historical dimension and therefore more or less mentally prepared for the visiting experience. Empirical studies found that the main motive of visitors of so-called dark heritage sites is a desire to understand the historical events, circumstances, and causes (Biran et al., 2011; Yankholmes and McKercher, 2015). Furthermore, Biran et al. (2011) found a positive relation between the perception of the site as closely related to personal family history and the visitor's motive for an emotional experience.

In contrast, at an everyday place people may at first be unaware of its historical dimension and stumbling stones or interpretation panels may either remain unnoticed or lead only to a casual recognition of the place's history.

In two empirical studies we addressed the affective impact of an everyday place's historical dimension on visitors. We found a site holding a ferocious history which is hardly known by the public: a former women's clinic where hundreds of forced sterilizations took place during the time of NS dictatorship. The building got completely renovated and today houses a research institute. We systematically manipulated the awareness of the building's history by the type of prior information given to participants. The aim of the two studies was to investigate the impact of history awareness on the affective personal level, the perception and evaluation of related information materials, and on the site itself.

### Personal Affect

There is empirical evidence that emotional engagement plays an important role when visiting dark heritage places (or otherwise engaging with dark history), as it may help visitors to generate meaning (Sigala and Steriopoulos, 2021) and to increase historical understanding, especially historical empathy (Savenije and de Bruijn, 2017). Usually, visitors report a high emotional involvement while being at a dark heritage site (Biran et al., 2011; Nawijn and Fricke, 2015; Bilewicz and Wojcik, 2018). While visitors most probably experience negative feelings when visiting a dark heritage site (Biran et al., 2011; Nawijn and Fricke, 2015; Bilewicz and Wojcik, 2018) or expect those feelings to be elicited during their visit (Nawijn et al., 2016, 2018), the spectrum of possible experienced emotions has been found to have a broad range, also including positive (e.g., pride, satisfaction, and hope) and mixed valence emotions (e.g., compassion, and awe; Nawijn et al., 2016, 2018; Oren et al., 2021). Brown (2015) found that on a cognitive level, tourists visiting memorials of the victims of Nazism increased their knowledge about NS history, while on an emotional level, they experienced feelings of sadness, shock, anger, despair, and incomprehension. Some tourists were overwhelmed by the affective experience and found it hard to resume the role of a tourist after their visit to the memorial. Nawijn and Fricke (2015) found that visitors of the KZ memorial Neuengamme experienced intense negative emotions but also developed behavioral intentions to revisit the site. The authors discuss that the negative emotions associated with the visit may give the proactive motivation for (long-term) behavioral consequences. In a later work, Nawijn and Biran (2019) resurrect the idea that negative emotions, which are “an integral part of the tourist experience” (p. 2386) at dark heritage places, may foster positive outcomes, such as eudaimonic experiences, and may even include a transformation of the self. However, Zheng et al. (2020) observed that negative emotions, such as sorrow, shock, and depression, experienced when visiting a dark heritage place may only indirectly result in spiritual meaning due to the mediating effect of the learning benefit. Regarding the emotion ‘fear', they neither found a direct nor an indirect impact on spiritual meaning.

In a longitudinal study Bilewicz and Wojcik (2018) investigated the emotional reactions of high school visitors of the KZ memorial Auschwitz and found the syndrome of secondary traumatic stress among 13.2% of them. The authors emphasize the importance of intense elaborations of Holocaust history and proper psychological preparations before the visit to places related to traumatic past events.

One reason for the extremely strong emotional reactions to dark heritage sites may be that visitors are not only aware of their historical dimension but also experience them as authentic places resembling the historic situation both in terms of appearance and atmosphere, described either by objective parameters such as size, lightning, spectrum of color, or by more abstract qualities like legibility, coherence, complexity, and mystery (Kaplan and Kaplan, 1989). In contrast, everyday places having a dark heritage background often lack a strong visual resemblance to the former historical situation. Therefore, emotional reactions should be primarily caused by becoming aware of the place's historical dimension and to a much lesser degree by its current visual appearance.

### Effect of Mood on the Evaluation of Further Information

Affect, along with cognition and conation, is one of the three components of the mind. Affect subsumes any experience of feeling, emotion, or mood. Mood has been defined as a short-lived affective state, which is usually of low intensity. However, a certain mood can last for hours up to weeks typically without the person knowing what prompted it (cf. American Psychological Association, 2022). It has been postulated that the personal affective state has influence on informational effects and on processing effects (Forgas, 2013). Affect congruence would be an informational effect, that is, when the affective state influences the valence of responses. This could be explained by the feelings-as-information approach from Schwarz (2001) postulating that people consciously or unconsciously use their mood as a source of information. Thus, their own negative mood is interpreted in such way that one finds oneself in a problematic situation, thereby influencing in turn the evaluation of stimuli in that situation. Accordingly, it has been shown that prior positive, negative, or neutral information can affect the subsequent emotional experience of pictorial stimuli (Wu et al., 2012).

A processing effect would be when affect influences the way information is processed, like the usage of different processing strategies. For example, two well-investigated processing styles are the bottom-up and the opposing top-down style. A bottom-up processing style describes that the incoming stimulus data initiates the processing of information, while on the other hand a top-down processing style describes that “an overall hypothesis about or general conceptualization of a stimulus is applied to and influences the analysis of incoming stimulus data” (cf. American Psychological Association, 2022). According to Bless and Fiedler (2006) moods signal whether a situation holds assimilative opportunities or accommodative challenges to the self. Accordingly, subconscious positive mood has been found to enhance a top-down processing style characterized by heuristic thinking, global focus, little attention for details, and a search for similarities, whereas negative mood enhances a bottom-up processing style characterized by analytical thinking, local focus, attention for details, and a search for differences (Forgas, 2013; Huntsinger, 2013; Bless and Burger, 2017).

Based on these findings, we assume that the negative mood induced by the awareness of being at a place with negative history should influence the evaluation (affect congruent judgments) and description (bottom-up processing style) of further information material.

### Perception and Evaluation of the Place Itself

In contrast to the effects of a place's atmosphere, few studies have dealt with the cognitive and affective effects of one's awareness of the historical dimension of a place itself (Lewicka, 2005, 2008; Baron, 2012; Blaison and Hess, 2016). Blaison and Hess (2016) found that a real threat (landfill) influenced participants' evaluations of places in the same way as a bygone and therefore historic threat (house, in which a murder took place 20 years ago): participants would pay less rent for a flat, which was near the threat. If the spacing to the threat passed a critical spatial distance, the effect inverted, and participants were willing to pay even more for a flat which was far away from the threat compared to an area with no threat at all. These effects are indicative of contagion, which describes the essentialist belief that attributes of humans and their activities (for example, crimes) transfer to objects or places that have been in contact with that person or have been used in the activity (Rozin et al., 1986). A related mechanism, termed emotional residues, has been reported by Savani et al. (2011). In a series of studies, they showed that a large proportion of participants believed that traces of previous emotions accumulate in physical spaces, thereby transferring affective states to subsequent visitors. Accordingly, participants tended to prefer to fill out a questionnaire in a room in which previous participants had experienced positive emotions rather than negative emotions. Taken together, these findings indicate that even in the absence of an authentic historic atmosphere, the awareness of its history may exert an influence on the visitor's experience of a site, particularly regarding the affective dimension.

## Research Questions and Hypotheses

In two empirical studies, we investigated the impact of becoming aware of an everyday place's NS history. We expected that the history awareness should induce effects on all of our dependent variables, namely, participant's mood and arousal, the affective evaluation (Study 1) and description (Study 2) of photographic material related to NS history, and the affective evaluation of the site itself. History awareness was manipulated by providing participants different kinds of prior information: the history and place awareness group (HP) received information about NS crimes in general combined with the information about the particular NS history of the building, the history awareness group (H) just received information about NS crimes in general, and the control group (C) received eighter no information at all (Study 1) or general information about the building's function today (Study 2).

It has been shown that the visit of a place with a tragic history influences visitor's mood in a negative way (Nawijn and Fricke, 2015). Therefore, we expected that participants of group H, should report more negative mood, compared to the control group C. Furthermore, participants of group HP should experience even more negative mood, compared to both groups H and C. Regarding the participant's arousal we expected the same pattern.

Furthermore, it has been shown that negative mood fosters affect congruent responses (Forgas, 2013). Therefore, in Study 1, in which the task was to rate the valence and arousal evoked by each of 80 NS related photos, we expected that group H should rate the photos more negatively and with higher evoked arousal compared to group C. Furthermore, group HP should rate the photos even more negatively and with even higher evoked arousal compared to both groups H and C.

Also, there is evidence that negative mood induces a bottom-up processing style which fosters attention to details (Forgas, 2013; Bless and Burger, 2017). In Study 2, the task was to describe three NS related photos, we expected differences regarding the level of detail in the descriptions of the different groups. A more detailed description could be indicated by naming more photo elements and a higher overall word count. We expected that group H's descriptions should include more photo elements and consist of higher overall word count compared to group C. Furthermore, we expected that group HP's descriptions should include even more photo elements and consist of a higher overall word count compared to both groups H and C.

Finally, based on studies showing that prior information about the negative history of a place affects the evaluation of the whole area (Blaison and Hess, 2016), we expected that group HP should rate the room in which the studies took place more negatively and with higher evoked arousal compared to both groups H and C.

## Study 1

### Methods

Both Studies were approved by the local institution's ethics committee. Participants were invited via email based on the institute's database of study volunteers. Participants who took part in Study 1 were not invited to participate in Study 2. All participants gave their informed consent before taking part in the studies and received eight euro for participation. At the end of each study, a debriefing took place.

#### Participants

A total of *n* = 108 participants took part in the study. Seven participants were excluded, three failed the manipulation check, three due to lack of language skills, and one due to technical problems that led to data loss. The final sample included 101 participants (C = 34, H = 33, HP = 34) between the ages of 18 and 31 (*M* = 22.95, *SD* = 2.75; 81 women).

#### Study Setting

The study took place in a former gynecological clinic, built in 1890. During the time of NS dictatorship at least 655 so-called “eugenic” sterilizations were conducted in the building.^2^ The buildings function as women's clinic ended in 2002. Extensive renovations lasted until the year 2011 when subsequently a department of the university and a research institute moved in. Today, as the building houses several laboratory rooms, appointed with modern equipment, one can hardly imagine its history.

#### Design

We used a single factorial between-subjects design with three conditions differing in their received prior information. To check participants' prior knowledge (manipulation check), two questions were queried at the end of the study: “Did you know beforehand that the building in which you currently are is a former women's clinic?” and “Did you know beforehand that in the time of NS regime in the former women's clinic forced sterilizations took place?”. Exclusion criteria were adapted to the contents of the groups' received prior information.

#### Materials

The independent variable was the (non-)presence of prior information. While group C did not receive any prior information, the groups H and HP received prior information *via* two different audio texts. Both audio texts were read by the same female speaker, consisted of 221 words, and had a total length of 2:42 mins. Group H‘s audio text offered general information about the various crimes authorized by the NS regime, namely, the Holocaust, the persecution of people with different political views, the murder of people who were physically or mentally challenged, as well as the forced sterilizations carried out on women who were classified as “racially inferior.” Group HP's audio text offered the same general information about the NS crimes as provided to group H, complemented with information about the building‘s NS history, namely, that it used to be a women's clinic where hundreds of forced sterilizations took place. To keep the duration of both audio texts similar, group H's text began by briefly mentioning parts of the instruction that all participants had received before in written form.

Each participant was asked to evaluate 80 *historical photographs*. Each of the photos dated back to the period of the NS regime and showed scenes associated with clinics and hospitals. The photos were gathered from the local university's archives and from research on the internet, and presented achromatic and either in a fixed horizontal (560 × 450 px) or vertical format (450 × 560 px) with a short caption describing its content and the approximate year it was taken.

#### Measures

To rate their mood and arousal (at 3 points in time) participants were asked “In what kind of mood are you in right now?” along with a nine-point Likert-scale extending from “very negative” to “very positive.” Analogously, to rate their arousal, participants were asked “How aroused/activated are you right now?” along with a nine-point Likert-scale extending from “not aroused at all” to “very aroused.”

To evaluate the 80 photos, participants were presented each photo and its caption along with the question “What kind of emotions does the picture evoke in you?” and the corresponding Self-Assessment Manikin (SAM; Lang, 1980; Bradley and Lang, 1994) valence rating scale, followed by the question “What is the extent of arousal that the picture evokes in you?” and the corresponding SAM arousal rating scale.

To categorize the photos, participants were given two tasks. First, they had to interpret each photo's content by choosing one or more of the following multiple-choice answers: “victim,” “offender,” “person neither victim nor offender,” “building,” “room” and “other.” In order to gain a comparable unit revealing possible differences between the interpretation of the 80 photos of the different groups (C, H and HP) we calculated the mean overall selection of each category within each of the different groups, with a possible range between 0 and 80. Second, participants had to rate which group of persons was put into the photo's focus of attention on a nine-point Likert-scale ranging from “definitely offender” to “definitely victim.”

#### Procedure

**Figure 1:**
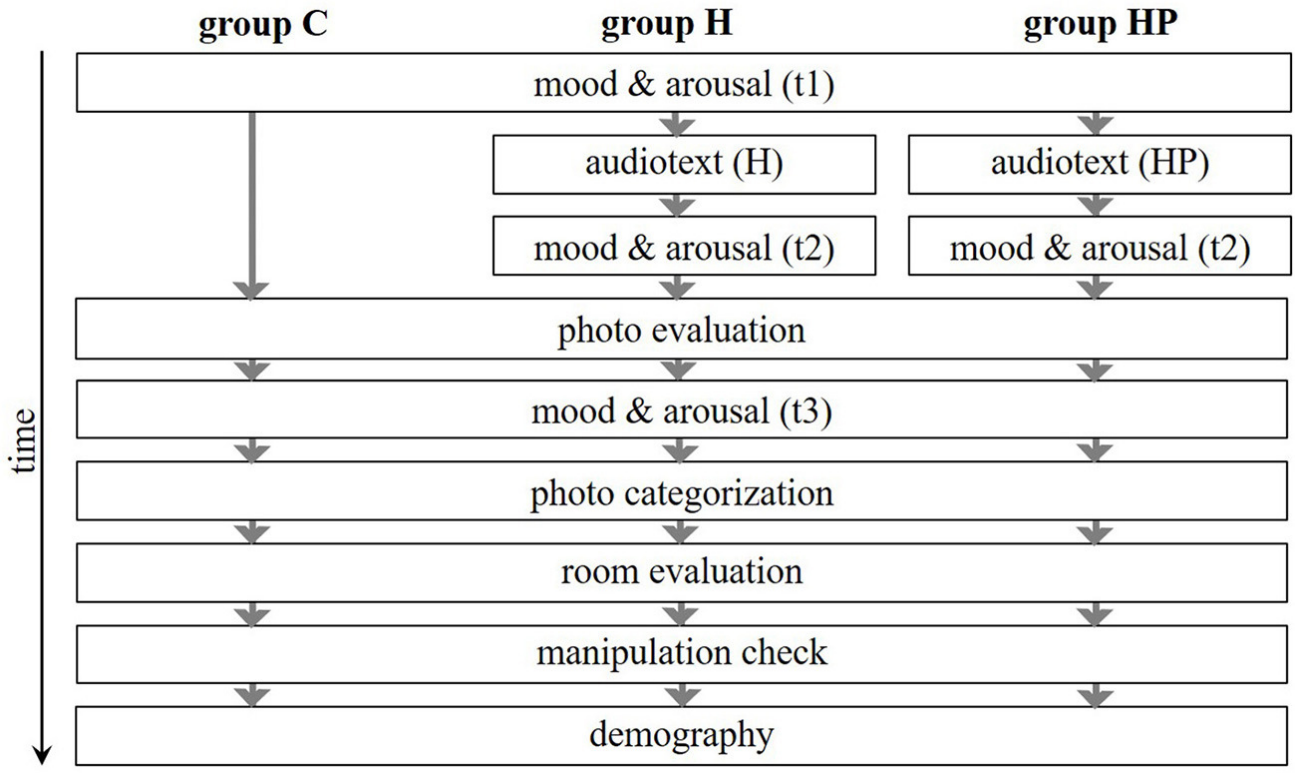
Procedure of Study 1. C, control group; H, history awareness group; HP, history and place awareness group.

The experimental room was equipped with a chair and a table on which s vertically positioned computer screen, a keyboard, a mouse, and a headphone were positioned. Participants were randomly assigned to one of the three conditions and received information about the procedure and the instructions for the study task. The first task was to rate their current mood and arousal (t1). Groups HP and H then heard an audio text and subsequently had to rate their current mood and arousal again (t2). The photo evaluation task began with an example to familiarize participants with the rating procedure. The 80 photos were presented randomly, and participants had to rate the valence and arousal evoked of each photo while watching it. Participants were asked to rate their current mood and arousal (t3) again. Afterwards the photo categorization took place, whereby the photos were presented randomly. Afterwards, participants had to evaluate the room, and the manipulation check, and the query of some demographic variables took place. The procedure is visualized in Figure 1.

### Results

#### Mood

To compare the personal mood of all three groups, we conducted a 3 × 2 Mixed Model ANOVA with the between-subjects factor condition (C vs. H vs. HP) and the within-subjects factor time (t1 vs. t3). The ANOVA revealed a main effect for condition, *F*_(2, 98)_ = 3.472, *p* < 0.05, η_*p*_^2^ = 0.066, indicating that the mood of group HP was more negative than that of group C. Further, there was main effect for time, *F*_(1, 98)_ = 345.224, *p* < 0.001, η_*p*_^2^ = 0.779, indicating that mood was significantly more negative at t3 than at t1. The ANOVA indicated no interaction between condition and time, *F*_(2, 98)_ = 0.678, *p* = 0.510, η_*p*_^2^ = 0.014. Descriptive data of mood and arousal is shown in Table 1.

**Table 1:** Participants' mood and arousal at three different times (t1, t2, t3) by condition.

		**Mood**		**Arousal**	
		***M***	***SD***	***M***	***SD***
C	t1	6.85	1.21	5.29	1.73
	t2	–	–	–	–
	t3	4.15	1.52	5.76	1.76
H	t1	6.64	1.75	5.03	1.76
	t2	4.67	1.51	4.88	1.58
	t3	3.76	1.30	5.39	1.87
HP	t1	6.32	1.45	5.29	2.02
	t2	4.15	1.44	5.62	1.65
	t3	3.18	1.22	6.24	1.76

*M, means; SD, standard deviations; C, control group; H, history awareness group; HP, history and place awareness group; lower numbers indicate negative mood/low arousal*.

**Figure 2:**
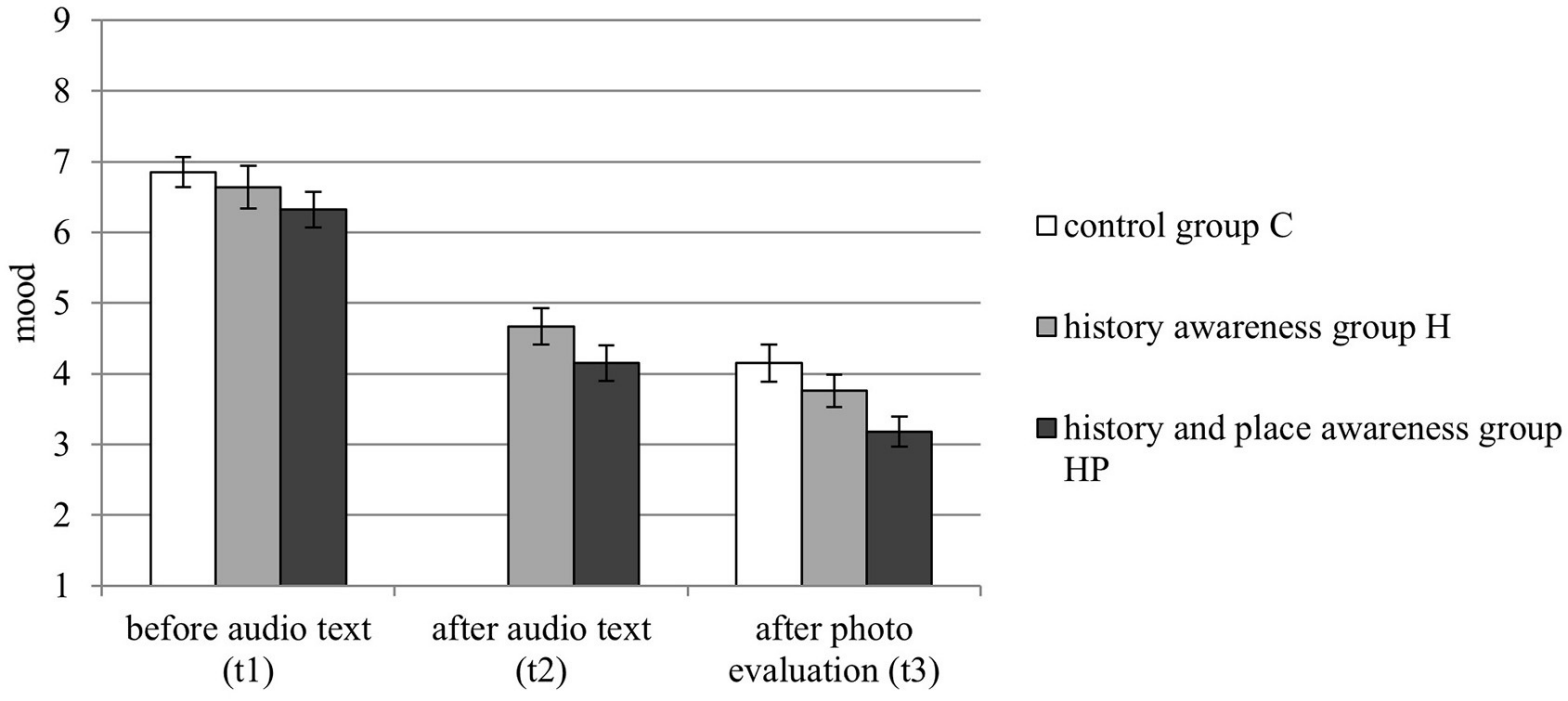
Participants' personal mood and arousal at three different times (t1, t2, t3) by condition. Lower scores indicate a more negative mood. Error bars represent standard error of mean.

To compare the mood of the groups H and HP, we conducted a 2x3 Mixed Model ANOVA with the between-subjects factor condition (H vs. HP) and the within-subjects factor time (t1 vs. t2 vs. t3). The ANOVA revealed no main effect for condition, *F*_(1, 65)_ = 2.768, *p* = 0.101, η_*p*_^2^ = 0.041, but again a main effect for time, *F*_(2, 130)_ = 138.149, *p* < 0.001, η_*p*_^2^ = 0.680, indicating that mood became more negative over time. Further, the ANOVA indicated no interaction between condition and time, *F*_(2, 130)_ = 0.287, *p* = 0.751, η_*p*_^2^ = 0.004. The ratings of mood at the different times are visualized in Figure 2.

#### Arousal

To compare the arousal of all three groups, we conducted a 3 × 2 Mixed Model ANOVA with the between-subjects factor condition (C vs. H vs. HP) and the within-subjects factor time (t1 vs. t3). The ANOVA revealed no main effect for condition, *F*_(2, 98)_ = 1.117, *p* = 0.331, η_*p*_^2^ = 0.022, but a main effect for time, *F*_(1, 98)_ = 8.752, *p* < 0.01, η^2^ = 0.082, indicating that participants' arousal significantly increased from t1 to t3. Additionally, the ANOVA revealed no interaction between condition and time, *F*_(2, 98)_ = 1.592, *p* = 0.458, η_*p*_^2^ = 0.016. Also, to compare the arousal of the groups H and HP, we conducted a 2x3 Mixed Model ANOVA with the between-subjects factor condition (H vs. HP) and the within-subjects factor time (t1 vs. t2 vs. t3). Again, the ANOVA revealed no main effect for condition, *F*_(1, 65)_ = 2.776, *p* = 0.100, η_*p*_^2^ = 0.041, but a main effect for time, *F*_(2, 130)_ = 6.318, *p* < 0.05, η_*p*_^2^ = 0.089, indicating that arousal both at t2 and at t3 was higher than at t1. Again, there was no interaction between condition and time, *F*_(2, 130)_ = 1.194, *p* = 0.306, η_*p*_^2^ = 0.018.

#### Evaluation of the Photos

To compare the photo evaluation of all three groups, we computed the mean overall valence and arousal regarding all 80 photos and conducted ANOVAs with the factor condition (C vs. H vs. HP). Descriptive data of the photo ratings are shown in Table 2.

**Table 2:** Participants' ratings of the 80 NS related photos by condition.

	**Valence**		**Arousal**	
	***M***	***SD***	***M***	***SD***
C	3.81	0.43	4.40	1.13
H	3.59	0.50	4.19	1.26
HP	3.45	0.71	4.72	1.30

*M, means; SD, standard deviations; C, control group; H, history awareness group; HP, history and place awareness group; lower numbers indicate negative valence/low arousal*.

The ANOVA indicated significant differences in the mean valence ratings due to condition, *F*_(2, 98)_ = 3.505, *p* < 0.05, η_*p*_^2^ = 0.067. Pairwise comparisons revealed that group HP (*M* = 3.45, *SD* = 0.71) rated the photos more negatively than group C (*M* = 3.80, *SD* = 0.43). Regarding the mean arousal ratings of the photos, the ANOVA indicated no significant differences due to condition, *F*_(2, 97)_ = 1.418, *p* = 0.247, η_*p*_^2^ = 0.028.

The photo categorization consisted of two tasks regarding the content and focus of each photo and was analyzed exploratively. To compare the ratings of the content of all three groups, we computed the mean overall selection of each category regarding all 80 photos and conducted ANOVAs with the factor condition (C vs. H vs. HP). Descriptive data of the photos' content categorization are shown in Table 3.

**Table 3:** Participants' interpretation of content of the 80 NS related photos by condition.

	**C**		**H**		**HP**	
	***M***	***SD***	***M***	***SD***	***M***	***SD***
Victim	37.29	7.33	39.73	7.28	40.56	8.22
Offender	23.88	9.83	26.76	8.88	30.62	9.88
Neither v nor o	28.82	9.88	23.75	9.65	23.70	9.67
Building	7.29	3.10	7.48	3.63	7.32	6.58
Room	19.88	8.27	18.91	8.27	18.35	9.14
Other	6.67	6.31	2.00	1.81	4.79	5.96

*M, means; SD, standard deviations; C, control group; H, history awareness group; HP, history and place awareness group, Neither v nor o, Neither victim nor offender*.

The ANOVA indicated significant differences in the mean selection of “offender”, *F*_(2, 98)_ = 4.260, *p* < 0.05, η_*p*_^2^ = 0.080. Pairwise comparisons revealed that group HP (*M* = 30.62, *SD* = 9.88) chose “offender” significantly more often than group C (*M* = 23.88, *SD* = 9.83). Further, the ANOVA indicated significant differences in the mean selection of “person neither victim nor offender”, *F*_(2, 96)_ = 3.248, *p* < 0.05, η_*p*_^2^ = 0.063. Descriptively, group C chose this category more often than both other groups, but none of the pairwise comparisons was significant. There were no differences in the mean selection of the categories “victims”, *F*_(2, 98)_ = 1.680, *p* = 0.192, η_*p*_^2^ = 0.033, “building,” *F*_(2, 98)_ = 0.016, *p* = 0.984, η_*p*_^2^ = 0.000, “room,” *F*_(2, 98)_ = 0.277, *p* = 0.277, η_*p*_^2^ = 0.006, and “other”, *F*_(2, 36)_ = 2.148, *p* = 0.131, η_*p*_^2^ = 0.107.

To compare the ratings of the focus of all three groups, we computed the overall mean rating of all 80 photos and conducted ANOVAs with the factor condition (C vs. H vs. HP). The ANOVA indicated no differences in perception of focus between the groups, *F*_(2, 98)_ = 2.506, *p* = 0.087, η_*p*_^2^ = 0.049.

#### Evaluation of the Room

Due to a technical problem, one participant could not rate the room. The sample included in the following analyses contained 100 participants (group C = 33, group H = 33, group HP = 34). Descriptive data of participants' ratings of the room are shown in Table 4.

**Table 4:** Participants' ratings of the room by condition.

	**Valence**		**Arousal**	
	***M***	***SD***	***M***	***SD***
C	3.36	1.03	4.67	1.85
H	3.85	1.03	4.24	1.90
HP	3.06	1.20	5.91	1.64

*M, means; SD, standard deviations; C, control group; H, history awareness group; HP, history and place awareness group; lower numbers indicate negative valence/low arousal*.

To test our hypotheses of perceived valence and arousal evoked by the room, we conducted ANOVAs with the factor condition (C vs. H vs. HP). Regarding the valence ratings of the room, the ANOVA indicated significant differences due to condition, *F*_(2, 97)_ = 4.441, *p* < 0.05, η_*p*_^2^ = 0.084. Pairwise comparisons revealed that group HP (*M* = 3.06, *SD* = 1.20) rated the room more negatively than group H (*M* = 3.85, *SD* = 1.03). Regarding the arousal ratings evoked by the room, the ANOVA again indicated significant differences due to condition, *F*_(2, 97)_ = 7.816, *p* < 0.01, η_*p*_^2^ = 1.39. Pairwise comparisons revealed that group HP (*M* = 5.91, *SD* = 1.64) rated the room with a higher arousal than group H (*M* = 4.24, *SD* = 1.90) and group C (*M* = 4.67, *SD* = 1.85).

### Discussion

The aim of Study 1 was to investigate the impact of becoming aware of the NS history of a site on personal affect, the evaluation of NS related photos, and the evaluation of the experimental room itself. A general effect of time, but not of condition, on mood and arousal was found, with mood becoming more negative and arousal becoming higher after participants were confronted with NS related photos. Regarding the evaluation of the room, significant differences in the valence and arousal ratings due to condition were found. Group HP perceived the room more negative than group H, and group HP perceived a higher arousal evoked by the room compared to both groups H and C. Furthermore, group HP rated the NS related photos more negatively and interpreted the persons in the photos more often as offenders compared to group C.

As Study 1 revealed only partial evidence of the impact of history awareness on personal affect, Study 2 aimed to replicate and extend these findings. While the methodical approach in Study 1 was the evaluation of a large number of photos on quantitative scales based on a rather short viewing time for each photo, the methodical approach in Study 2 should be a more detailed description of individual photos in a qualitative way. Even though the analyses of the data solely with the female participants did not show any differences regarding the results, we decided to only invite female participants to Study 2. This decision was based on the aim to homogenize the sample and further eliminate potential gender biases, which could be substantiated within the study implications (victims at the site were women, while offenders were both men and women).

## Study 2

### Methods

#### Participants

A total of *n* = 99 participants took part in Study 2. Due to technical problems, participants actual ages could not be collected. However, we did collect participants year of birth (*M* = 1994, *SD* = 3.07), which ranged from 1983 to 1999. Eleven participants needed to be excluded, eight failed the manipulation check and three due to lack of language skills. The final sample included 88 participants (C = 30, H = 28, HP = 30); 87 indicated female, one indicated “other.”

#### Study Setting

The study setting was the same as in Study 1.

#### Design

We used a single factorial between-subjects design with three conditions differing in their received prior information. The same manipulation check and exclusion criteria as in Study 1 were used.

#### Materials

The independent variable was the type of prior information offered *via* three *audio texts*. The audio texts of groups H and HP were the same as in Study 1. The audio text presented to group C offered general information about the structural features of the building (total number of floors and rooms) and several facts about the research institute. All three audio texts fulfilled the same formal criteria as in Study 1.

**Figure 3:**
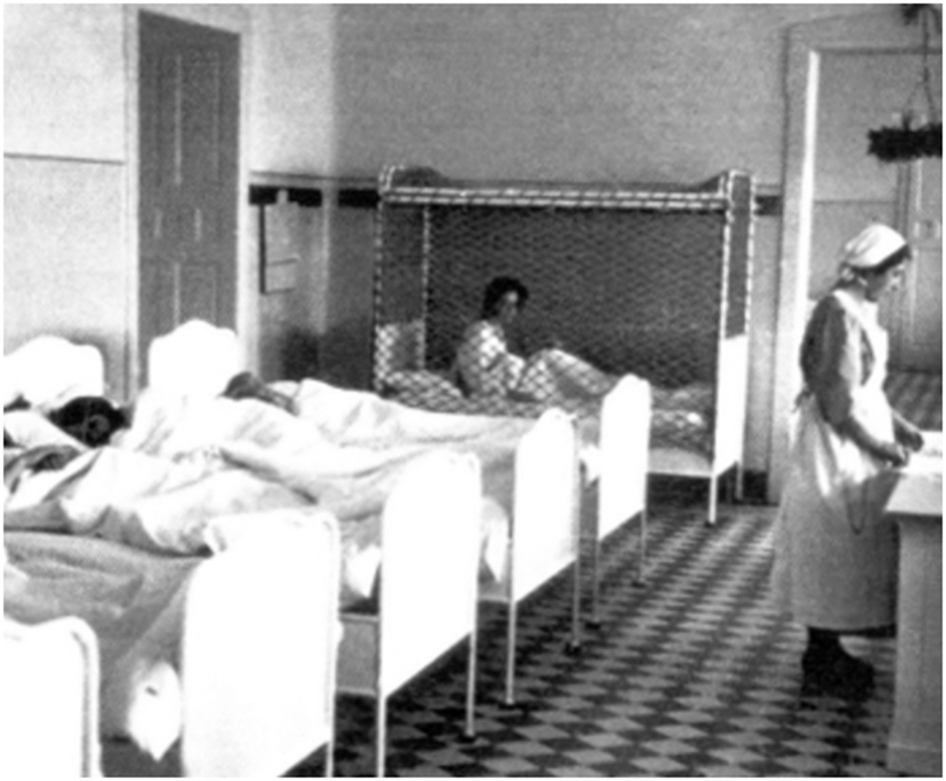
Photo 2. The photo's caption was “Hospital ward of a sanatorium, approx. 1935.” Source reference: DÖW, Foto-Signatur 7908.

Each participant was asked to verbally describe three *historical target photographs* (Photo 1, 2 and 3). The photos were presented in the same formal way as in Study 1, all of them in the fixed horizontal format. Photo 1 showed a nurse and two male patients lying in clinical beds. Its caption was “Nurse in a military hospital, approx. 1943.” Photo 2 showed a nurse and several beds arranged in a row with patients. One of the beds is surrounded by a cage. Its caption was “Hospital ward of a sanatorium, approx. 1935.” Photo 3 showed three men, two wearing jackets and one with a white doctor's coat, positioned at a table with documents. Its caption was “Deputy Reich Health Leader and Doctors, approx. 1938.” One of the photos is exemplarily shown in the following Figure 3.

We wanted to compare the ratings of personal affect with computer-based analyses of voice qualities, so we added a neutral Photo 0 and the default text “Northwind and Sun” (N&S). Photo 0 was chosen to serve as baseline for the nature of photo descriptions and showed a scene at a scientific exhibition without and reference to the time of NS dictatorship. N&S is a fable attributed to Aesop, considered as emotional neutral. Participants had to read N&S aloud before and after the audio text. As the analyses of the computer-based voice characteristics turned out to be technically too difficult, we will not report these results here.

#### Measures

**Figure 4:**
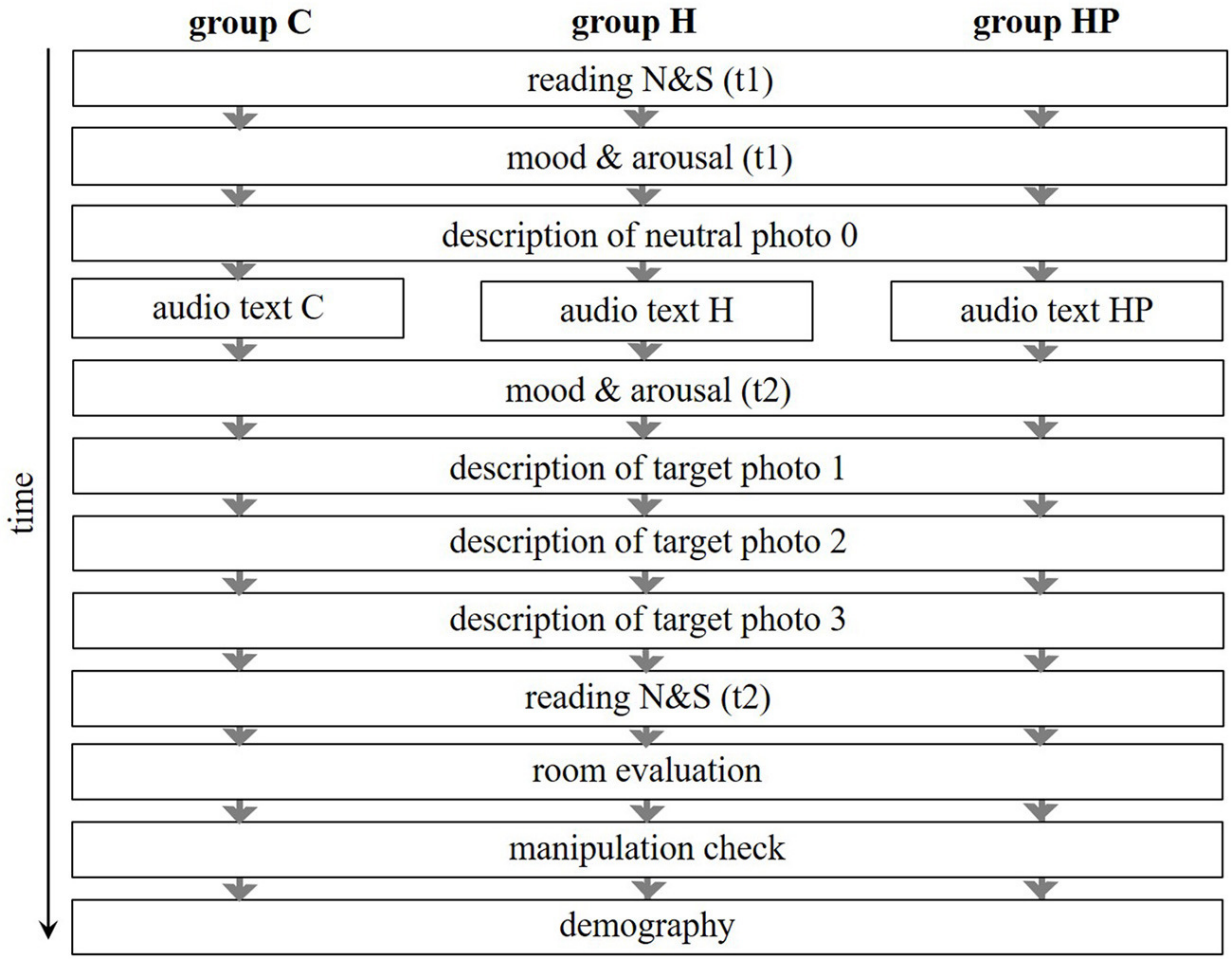
Procedure of Study 2. C, control group; H, history awareness group; HP, history and place awareness group.

The same measurements as in Study 1 were used for the rating of mood and arousal and the evaluation of the room. Furthermore, participants had to read N&S aloud at 2 points in time (see Figure 4).

Participants were asked to describe each photo in their own words within a minimum of 40 sec and no upper time limit. Descriptions were given verbally and recorded by a headset microphone. Later, each audio file was transcribed by a student assistant and its total word count was calculated via *Linguistic Inquiry and Word Count* (LIWC; Pennebaker et al., 2015). To systematically gather the number of named photo elements, we developed a coding system. The total possible number of named photo elements was 11 in photo 1, 16 in photo 2, and 10 in photo 3. Two student assistants blind to condition coded the photo elements via MAXQDA Plus 2018 (Release 18.0.3). The interrater reliabilities of the coders on each photo were estimated via SPSS Macro KALPHA by Hayes and Krippendorff (2007), Krippendorfs α _Photo1_ = 0.97 and Krippendorfs α _Photo2_ = 0.98. A third independent rater subsequently decided on the ratings that did not match.

#### Procedure

The beginning and end procedure of Study 2 were similar to Study 1. The same equipment as in Study 1 was used; except that the computer screen was positioned horizontally, and the headphone included a microphone. Participants were instructed to adjust their headset and read N&S aloud (t1). They then rated their mood and arousal (t1) and afterwards described Photo 0. The audio text was presented, and participants had to rate their mood and arousal again (t2). Afterwards, participants consecutively described photo 1, photo 2 and photo 3, and again read aloud N&S (t2).

### Results

#### Mood

To compare the mood of all three groups, we conducted a 3x2 Mixed Model ANOVA with the between-subjects factor condition (C vs. H vs. HP) and the within-subjects factor time (t1 vs. t2). Descriptive data of mood and arousal is shown in Table 5.

**Table 5:** Participants' mood and arousal at two different times (t1, t2) by condition.

		**Mood**		**Arousal**	
		***M***	***SD***	***M***	***SD***
C	t1	6.03	1.77	5.20	1.75
	t2	5.63	1.50	4.60	1.87
H	t1	5.96	1.43	4.79	2.01
	t2	3.82	1.16	5.32	1.85
HP	t1	6.23	1.33	4.90	1.79
	t2	3.20	1.30	5.13	1.81

*M, means; SD, standard deviations; C, control group; H, history awareness group; HP, history and place awareness group; lower numbers indicate negative mood/low arousal*.

**Figure 5:**
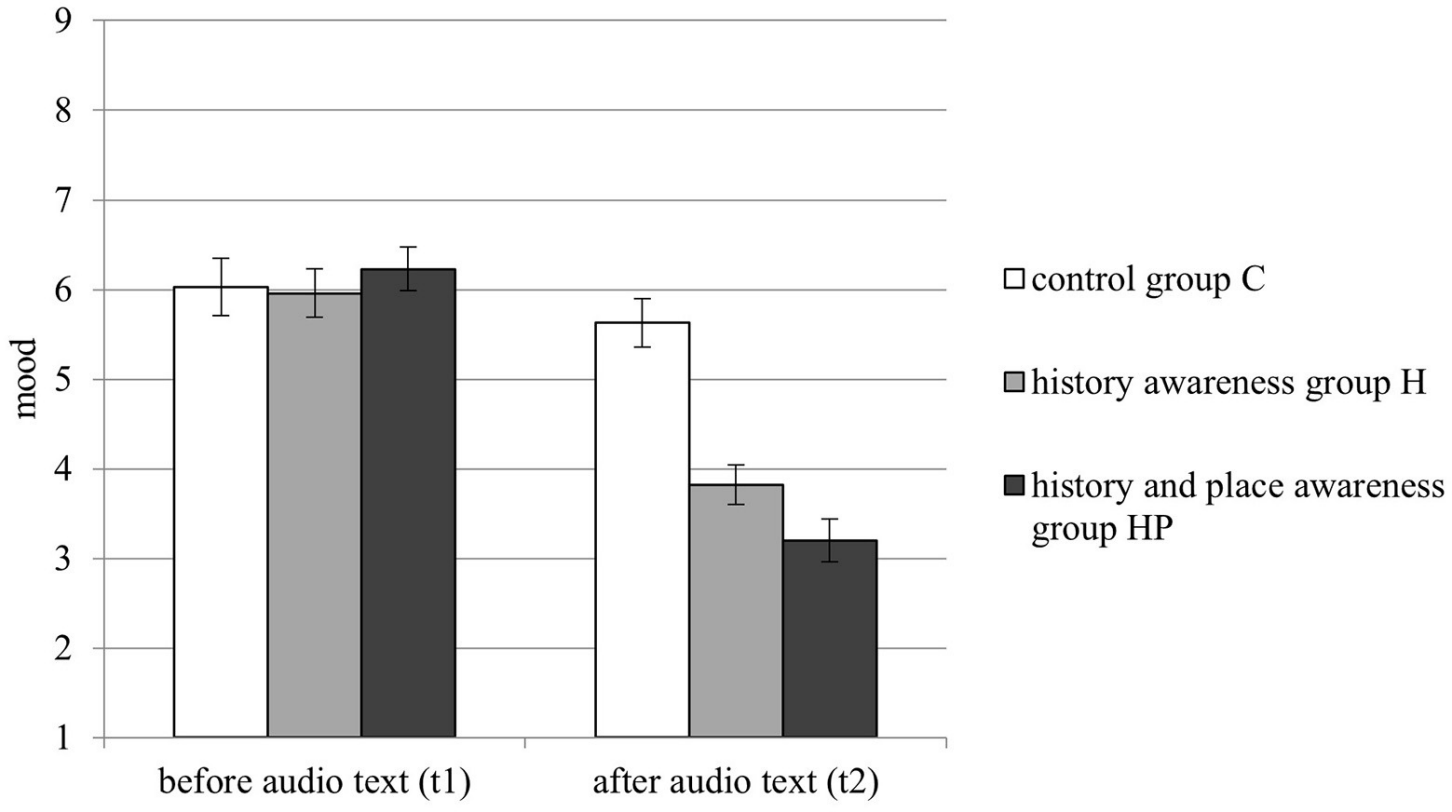
Participants' personal mood and arousal at three different times (t1, t2, t3) by condition. Lower scores indicate a more negative mood. Error bars represent standard error of mean.

The ANOVA indicated a main effect for condition, *F*_(2, 85)_ = 7.664, *p* < 0.01, η_*p*_^2^ =*0.1*53, with pairwise comparisons revealing that the mean mood over both times was significantly more negative in groups H and HP compared to group C. Also, the ANOVA indicated a main effect for time, *F*_(1, 85)_ = 118.639, *p* < 0.001, η_*p*_^2^ = 0.583, revealing that mood was significantly more negative at t2 than at t1. In addition, there was a significant interaction between condition and time, *F*_(2, 85)_ = 20.993, *p* < 0.001, η_*p*_^2^ = 0.331, indicating that mood did not differ between conditions at t1 but was significant more negative for group HP and group H than for group C at t2. However, at t2 there was no significant difference of mood between the groups H and HP. Mood ratings at the different times are visualized in Figure 5.

#### Arousal

To compare the arousal of all three groups, we conducted a 3x2 Mixed Model ANOVA with the between-subjects factor condition (C vs. H vs. HP) and the within-subjects factor time (t1 vs. t2). The ANOVA indicated neither a main effect for condition, *F*_(2, 85)_ = 0.074, *p* = 0.928, η_*p*_^2^ =*0.0*02 nor a main effect for time, *F*_(1, 85)_ = 0.089, *p* = 0.767, η_*p*_^2^ = 0.00, but a significant interaction between condition and time, *F*_(2, 85)_ = 3.223, *p* < 0.05, η_*p*_^2^ = 0.070. Descriptively, arousal of group C decreased while the arousal of groups H and HP increased from t1 to t2, but none of the pairwise comparisons was significant.

#### Description of the Photos

Due to technical problems some audio recordings broke off before participants finished their photo description. The broken files were excluded from further analyses. Regarding photo 1 one file was excluded, resulting in the analyses of *n* = 87 (C = 30, H = 28, HP = 29) files. Regarding photo 2, 15 files were excluded resulting in the analyses of n=73 (C = 27, H = 21, HP = 25) files. Regarding photo 3, 37 files were excluded. By virtue of this huge data loss, we decided to leave photo 3 out of further analyses. To compare the word counts and numbers of named photo elements between the three groups, we conducted ANOVAs with the factor condition (C vs. H vs. HP).

##### Photo 1

The ANOVA indicated no differences in the mean word counts between group C (*M* = 132.70, *SD* = 63.75), group H (*M* = 161.71, *SD* = 87.82), and group HP (*M* = 138.62, *SD* = 69.05), *F*_(2, 84)_ = 1.230, *p* = 0.298, η_*p*_^2^ = 0.028. Also, there were no differences in the mean named photo elements between group C (*M* = 7.90, *SD* = 1.40), group H (*M* = 7.25, *SD* = 1.80), and group HP (*M* = 7.97, *SD* = 1.32), *F*_(2, 84)_ = 1.938, *p* = 0.150, η_*p*_^2^ = 0.044.

##### Photo 2

The ANOVA indicated no differences in the mean word counts between group C (*M* = 114.07, *SD* = 43.13), group H (*M* = 126.24, *SD* = 66.42), and group HP (*M* = 100.76, *SD* = 33.35), *F*_(2, 70)_ = 1.598, *p* = 0.209, η_*p*_^2^ = 0.044. Also, there were no differences in the mean named photo elements between group C (*M* = 8.52, *SD* = 2.86), group H (*M* = 7.48, *SD* = 2.38), and group HP (*M* = 7.16, *SD* = 1.72), *F*_(2, 70)_ = 2.310, *p* = 0.107, η_*p*_^2^ = 0.062.

#### Evaluation of the Room

To test our hypotheses of perceived valence and arousal evoked by the room, we conducted ANOVAs with the factor condition (C vs. H vs. HP). While descriptively, the pattern resembled Study 1, no significant differences in valence ratings of the room were found between the groups C (*M* = 3.90, *SD* = 0.84), H (*M* = 3.75, *SD* = 1.32), and HP (*M* = 3.47, *SD* = 1.43), *F*_(2, 85)_ = 0.968, *p* = 0.384, η_*p*_^2^ = 0.022. Also, while descriptively similar to Study 1, there were no differences in perceived arousal evoked by the room between the groups C (*M* = 4.57, *SD* = 2.08), H (*M* = 5.07, *SD* = 1.90), and HP (*M* = 5.57, *SD* = 1.68), *F*_(2, 85)_ = 2.093, *p* = 0.130, η_*p*_^2^ = 0.047.

### Discussion

The aim of Study 2 was to replicate and extend the findings of Study 1. An effect of condition and time on mood and, furthermore, an interaction of condition and time on mood and arousal were found. In the groups H and HP mood became more negative after the audio text compared to group C. Also, arousal of the groups H and HP became descriptively higher after the audio text compared to group C, but pairwise comparisons revealed no significant differences. Regarding the descriptions of photos 1 and 2, there were neither significant differences in the mean overall word count nor in the mean number of named photo elements due to condition. The evaluation of the room descriptively showed the same pattern as in Study 1, but differences between the groups were not significant.

## General Discussion

The present studies aimed to investigate the effects of becoming aware of a place's NS history on visitors' personal affect, the evaluation (Study 1) and description (Study 2) of related pictorial documents, and on the evaluation of the site itself.

An extensive number of studies within a range of disciplines have investigated visitors' motives and emotions at KZ memorial sites, but less is known about these processes at everyday places holding non-salient historical dimensions. Our studies took up this issue by disentangling the effect of history awareness from the effect of a site's current readability.

In general, we found that being confronted with information and pictorial material related to the NS period led to a more negative mood in both studies. There was also a larger decrease in mood for those groups that received information about NS crimes prior to the presentation of the photos, compared to the control group in Study 2, while no such differences were found in Study 1. One reason for this difference between the studies may be the number of photos presented. In Study 1, participants were shown 80 photos, which alone may have led to a substantial decrease in mood, whereas in Study 2, only three photos were shown, which may have led to a decrease in mood only in concert with additional information about the NS crimes. However, no evidence was found that the decrease in mood was particularly pronounced when participants became aware of being at a place where NS crimes happened. Similarly, arousal in all three groups increased after the evaluation of the 80 photos in Study 1, whereas Study 2 found an interaction between time and condition, but pairwise comparisons did not show significant differences between the groups. Descriptively the data showed that participants of groups H and HP reported higher arousal after receiving the prior information, whereas arousal in the control group decreased. Again, no evidence was found that the increase in arousal was particularly pronounced when participants became aware of being at a place where NS crimes happened.

These findings stand in contrast to previous studies conducted at KZ memorial sites which report intense negative feelings of visitors (Biran et al., 2011; Brown, 2015; Nawijn and Fricke, 2015; Bilewicz and Wojcik, 2018). The main difference between the present and the previous studies lies in the visible features of the places: KZ memorial sites show and tell the NS crimes and make them tangible through the site's atmosphere, their architectural features, and authentic material artifacts. In contrast, the present studies were conducted at a seemingly neutral place giving no hints for the crimes that happened within it. As a tentative conclusion from the present studies' findings, it seems that besides becoming aware of the historical dimension of a place additional contextual cues play an important role regarding affective effects on visitors.

Further, we assume that the higher the personal involvement within a topic or the closer to the topic, the more pronounced the affective outcomes (Nawijn et al., 2018). If a person has only low involvement within the topic, we expect the smallest affective reaction. The personal involvement may be influenced by an interplay of various variables, including the awareness of a person to be somehow related to a victim or an offender, the person's age (temporal distance to the certain period in time), the person's geographic closeness, and the person's affiliation to a cultural community. As for our specific sample with an age ranging from 23 to 39 years, we assume the personal involvement be medium, as the participants did not experience the historical events directly but can rather be seen as the 3rd to 4th generation after the NS period.

Study 1 showed that the awareness of being at a site with NS history had a substantial effect on the evaluation of NS related photos. While there was no difference between the ratings of group H and HP, the latter rated the photos more negatively than participants of group C. This finding indicates that while there is no effect from prior information on the personal affective level, there may be an effect on the subsequent evaluation of further information material. Knowing about the crimes that happened at this particular site may have led participants to interpret and therefore evaluate associated pictures more negatively compared to the control group who may have interpreted the pictures differently and therefore rated their valences more neutrally.

Regarding the qualitative description of NS related photos, Study 2 showed no differences in overall word count or the amount of named photo elements due to prior information. Our hypothesis was that the negative mood (induced by the awareness of being at a site holding a NS history) would foster a bottom-up processing style, resulting in more detailed descriptions of the photos. As we found no differences in mood due to prior information, there may have been no different processing styles induced. However, because of data loss due to technical problems, these null results need to be interpreted with care. Nevertheless, the differences in attributing meaning to the photos found in Study 1 indicate that depending on prior information participants tended to interpret NS related photos differently. This is in line with the finding that group HP categorized the contents of the 80 pictures more often as “offender” compared to group C.

Finally, we investigated the impact of becoming aware of being at a historical site on the evaluation of the experimental room itself. Therefore, participants had to rate the valence and arousal evoked by the room. Study 1 found that group HP rated the room more negatively than participants of group C and with a higher evoked arousal than both groups H and C. These results are in line with previous findings showing that the awareness of a places' negative history influences the evaluation of the place (Savani et al., 2011) or a nearby place (Blaison and Hess, 2016) in a negative way. While descriptive data showed the same pattern in Study 2, differences between the groups did not show significance, indicating that the prior information as such was not enough to influence the evaluation of the room. However, prior information combined with further photographic material seems to affect the evaluation of the room but evaluating a larger number of respective pictorial stimuli seems to have a greater effect than the closer examination of few individual photos.

To the best of our knowledge, these are the first studies which investigated the effects of becoming aware of the dark history of an everyday place in an experimental design, thereby achieving a balance between ecological validity and methodological rigor. Nevertheless, there are certainly some empirical limitations. While affective reactions were determined via the two dimensions of mood and arousal, research at KZ memorial sites has shown that visitors experience a broad range of different emotions (Brown, 2015). Therefore, future studies should try to investigate the spectrum of possible affective reactions in more detail. Also, the present studies took place in a neutral room with no indication of its history and presumably no atmospheric resemblance to former NS times. In order to gain more insights into the interplay of a place's atmosphere and the awareness of its historic dimension, both aspects should be systematically crossed in future experiments.

Taken together, the affective effects of becoming aware of the historicity of a place were found to be small, at least in the present studies. Given that the room itself was perceived to be significantly more negative and evoked more arousal, we may assume that participants did indeed notice its NS crime history. Therefore, one may speculate that in order to exert some influence on visitors' mood and arousal, becoming aware of the history of a place is not sufficient enough in itself but has to be embedded in a context that reflects to a certain degree the authentic atmosphere of the historic place. Accordingly, KZ memorials typically show clearly visible material traces of its history and at least partly reproduce the original atmosphere, whereas the present studies took place in a neutral room with no visible signs of its history. Further research on this interplay of historical awareness and authentic atmosphere is needed in order to investigate whether the presence of an authentic atmosphere is the primary source of visitors' history-related feelings or whether the awareness of being at a historical place contributes further to the intensity of these feelings.

What should practitioners keep in mind regarding (guided) visits to everyday places holding a dark NS history? As such everyday places often appear to be inconspicuous, sometimes lacking physical remains, or their actual appearance does not resemble their former historical conditions anymore, the awareness of the historical dimension of such places needs to be induced explicitly. One option to do so is the usage of applications displayed on mobile devices (Harley et al., 2016; Price et al., 2016; Amakawa and Westin, 2017). The application may provide multimedia presentations or augmented reality (AR), making it possible to offer additional visual or auditive information (e.g., information about a place's former appearance, its former usage, and the background noise that typically occurred at the place in former times). Another way to promote students' or other visitors' learning about (local) history is to combine different learning opportunities, such as lectures about the past, visiting sites, and performing one's own historical research. It has been shown that these combined learning activities foster increased interest in that history (Stefaniak et al., 2017). However, when it comes to students visiting a historic place related to traumatic past events, it is advisable to elaborate this history and offer proper psychological preparation before the visit (Bilewicz and Wojcik, 2018).

### Conclusions

The present studies investigated the effects of history awareness at an everyday place holding a dark NS history on participants' affective and cognitive outcomes. Both studies showed an effect of history awareness on the participants' personal affect. However, the personal affective reactions of the participants did not differ much between the participants being aware of the NS history in general and those participants being aware of the NS history in general combined with the NS history of the building in which the studies took place. In conclusion, there is evidence that history awareness influences personal affect, but becoming aware of being at a place that was part of this history does not particularly pronounce this effect. This contrasts with studies conducted at memorial places that found far more intense emotional reactions of its visitors. Therefore, we suggest that the affective effects fostered by the visit to a historic place intensify when the place's physical atmosphere resembles this history to a certain extent. Considering this, preserving, and restoring historic places seems reasonable in order to enable visitors to experience the look and feel of the former times or to attain intensive feelings at the place. Also, regarding historic places, much material remains lacking; the opportunities offered by digital media should receive more attention.

In addition, it may be that history awareness influences the processing of related historic documents, but the present studies did not support this hypothesis. However, the findings regarding the photo description need to be interpreted with caution, as technical problems caused a substantial data loss.

Finally, the present studies found evidence that the awareness of being at a place where NS crimes happened influences the evaluation and interpretation of related historic photos and also found partial evidence for its influence on the evaluation of the place itself. These findings indicate that visiting a historic place and knowing about the crimes that happened there does make a difference regarding the perception of the place itself as well as the perception and interpretation of historic documents presented on site. We conclude that placing interpretation panels at historic places may be an appropriate tool to guide awareness toward the place's history and may additionally influence how an otherwise everyday setting is experienced. However, it should be noted that these interpretation panels probably work best when visitors have a certain amount of historical knowledge before the visit, which then becomes activated and actualized.

Taken together, the pattern of results found in the present studies suggests that even in the absence of a resembling physical atmosphere becoming aware of the historical dimension of a place may exert some affective influence on its visitors.

## Author's Note

Before marriage MR was named Melissa Gussmann and has previously published under the name Melissa Gussmann.

## Data Availability Statement

The raw data supporting the conclusions of this article will be made available by the authors, without undue reservation.

## Ethics Statement

The studies involving human participants were reviewed and approved by the Ethics Committee of the Leibniz-Institut für Wissensmedien, Tübingen. The patients/participants provided their written informed consent to participate in this study.

## Author Contributions

Study 1 and 2 conception and design, analysis and interpretation of results, and draft manuscript preparation: MR and SS. Data collection: MR. All authors reviewed the results and approved the final version of the manuscript.

## Conflict of Interest

The authors declare that the research was conducted in the absence of any commercial or financial relationships that could be construed as a potential conflict of interest.

## Publisher's Note

All claims expressed in this article are solely those of the authors and do not necessarily represent those of their affiliated organizations, or those of the publisher, the editors and the reviewers. Any product that may be evaluated in this article, or claim that may be made by its manufacturer, is not guaranteed or endorsed by the publisher.

## Footnotes

^1^cf. https://www.stolpersteine.eu/.

^2^cf. Bayer (2008).
